# Peers at work: Evidence from the lab

**DOI:** 10.1371/journal.pone.0192038

**Published:** 2018-02-06

**Authors:** Roel van Veldhuizen, Hessel Oosterbeek, Joep Sonnemans

**Affiliations:** 1 WZB Berlin, Berlin, Germany; 2 University of Amsterdam, Amsterdam, The Netherlands; 3 Tinbergen Institute, Amsterdam/Rotterdam, The Netherlands; Universidad de Alicante, ITALY

## Abstract

This paper reports the results of a lab experiment designed to study the role of observability for peer effects in the setting of a simple production task. In our experiment, participants in the role of workers engage in a team real-effort task. We vary whether they can observe, or be observed by, one of their co-workers. In contrast to earlier findings from the field, we find no evidence that low-productivity workers perform better when they are observed by high-productivity co-workers. Instead, our results imply that peer effects in our experiment are heterogeneous, with some workers reciprocating a high-productivity co-worker but others taking the opportunity to free ride.

## Introduction

This paper uses a lab experiment to improve our understanding of the nature of peer effects in the setting of a simple team production task. In particular, our experiment aims to shed light on the importance of observability. Do workers increase their effort when matched with a more productive co-worker? And does this effect depend on whether others can observe their productivity or whether they can observe the productivity of others? And are these peer effects similar for low-ability and high-ability workers?

Peer effects in team production can either be positive or negative. Workers can get motivated by high performance of colleagues, but it can also demotivate them or lead to free riding. Clearly, whether peer effects are positive or negative has consequences for the optimal organization of a workplace. Learning about the mechanisms that drive peer effects is therefore important.

A number of recent studies from the field and the lab have examined the presence of peer effects in the workplace. Whereas field studies may be higher in external validity, lab experiments can more easily avoid the reflection problem [1], allow for easier direct measurement of productivity, and may therefore have greater internal validity. [2] study peer effects in the workplace using a lab experiment with 24 high school students. The students had to spend four hours preparing the mailing for a questionnaire. Eight students worked alone, the other 16 worked at the same time and in the same room as one other student. [2] find evidence of peer effects in that the standard deviation in output within the two person groups is smaller than expected without peer effects, and that group output is higher than the output of workers in the individual treatment.

[3] use a data set on the productivity of cashiers (number of items checked per minute) of a large supermarket chain and show that cashiers in their supermarkets are influenced by the productivity of their co-workers. A key result of their study is that “worker effort is positively related to the productivity of workers who see him, but not workers who do not see him” (p. 112). They interpret this finding by arguing that social pressure can internalize free riding externalities, but social preferences seem to be irrelevant.

[4] had participants in the lab conducting a slider task in teams of three. The first member of the team was observed by the second, while the third worked without observing or being observed. The authors also vary the compensation scheme: individual piece-rate or team-based. The results are mixed: participants who are observed increase productivity when the compensation is team-based, but only in the first rounds. Observing team members react to what they see but only under the individual piece-rate regime. Other lab experiments that find positive peer effects in the workplace include [5–8].

A study that fails to find peer effects in the workplace is [9]. These authors study peer effects among professional golf players who are randomly paired to a partner. The results exclude effects as small as 0.043 strokes for a one stroke increase in playing partners’ ability. [9] speculate that peer effects may be absent in their setting because golf players are exposed to strong financial incentives and therefore already perform as well as they can. They further argue that professional golf players are perhaps selected on their ability to avoid the influence of playing partners.

[10] compare the results of lab experiments and field studies that investigate peer effects in the workplace and conclude that estimates from the lab generalize *quantitatively* to the field. This is a surprising and interesting result, given the large variation across the studies included in the sample. Of the 34 studies included in their analysis, 17 find significantly positive peer effects, 16 find no significant peer effects and one study reports significantly negative peer effects. Larger peer effects are reported in studies where a portion of compensation was determined by group output, where the workers were not perfect substitutes in production and where workers did not compete in the production process.

We contribute to the literature by using a novel design to study peer effects in a team production setting, focusing on the role of observability. Participants in our laboratory experiment work in teams of four. Each team of four has to jointly complete a fixed number of addition problems. The exact number of addition problems is never revealed. Team members are paid a fixed amount independent of their individual performance. A team is finished—can leave the lab—when it has completed the number of addition problems assigned to it. We then vary observability in each team of four by changing whether a participant can observe the cumulative number of addition problems solved by one of their team members. Before participants work in teams, they first work for four minutes individually on addition problems. This provides us with a measure of participants’ baseline productivity unaffected by any peer effects.

There are three between-subject treatments. In treatment “Baseline Productivity” (BaseProd, N = 84), participants are also informed about the baseline productivity of the other members in the team. In treatment “Contemporaneous Productivity” (ContempProd, N = 84), participants do not receive information about the baseline productivity of others. In treatment “No Peers” (N = 20), participants work individually and not in teams.

This design allows to test the following hypotheses:

- H1 (positive peer effects): A participant’s effort is increasing in the average baseline productivity of his/her team members.
- H2 (observability): A participant’s effort is increasing in the baseline productivity of team members that observe him/her. His/her effort is not affected by the baseline productivity of team members that he/she observes.
- H3 (ability): A low ability participant’s effort is more positively affected by the average baseline productivity of his/her team members than a high ability participant’s.

The first hypothesis reflects the average peer effect reported in [10], the second and third are based on results reported by [3]. In contrast to the findings of [2–4] but consistent with [9] (and 16 other studies surveyed by [10]) we find no support for H1. In contrast with the findings of [3] we also fail to find support for H2 and H3. Instead, additional analysis suggests that some workers reciprocate a fast co-worker but others take the opportunity to free ride, leading to an average null effect overall.

The remainder of this paper is structured as follows. The next section describes the details of the experimental design, the subsequent section presents and discusses the results and the final section concludes.

## Materials and methods

We designed an experiment that allows us to test hypotheses H1, H2 and H3. Because hypotheses H2 and H3 are inspired by the results from the field study of [3], our design attempts to capture some features of the work setting of the supermarket chain they analyzed. All data and code used to run the experiment and analyze the data are available from the Open Science Framework at https://osf.io/mv9eh/.

All participants in the experiment participated in two phases. In the first (baseline) phase, participants worked alone, allowing us to obtain a measure of their baseline productivity in the absence of peer effects. In the second (or production) phase, participants worked in teams of four (with the exception of treatment NoPeers, see below), allowing us to investigate the impact of peer effects. The baseline phase was identical for all participants, the production phase differed across the three (between-subject) treatments. In the remainder of this section we describe the two phases and discuss how the design allows us to test hypotheses H1 to H3.

The ethics committee of the Department of Economics and Business of the University of Amsterdam approved the experiments with human subjects reported in this study. The IRB granted approval based on the observation that the experiment adheres to the rules set by the Center for Research in Experimental Economics and political Decision making (CREED). No specific approval for specific experiments, such as ours, is required. CREED is a renowned institute for experimental economic research and adheres to the standards set in experimental economics. The collection, storage, protection, retention, and destruction of all data comply with national and EU regulations.

### Baseline phase

The experiment was computerized using PHP/MySQL. Participants in the experiment had to perform a production task that consisted of adding three two-digit numbers. We chose this task since it is easy to understand and mimics some features of the production process described by [3]—in particular its repetitive real effort nature. It also results in sizeable differences in productivity between participants, which allows us to examine differences between low-productivity and high-productivity workers. Arithmetic problems have been used as a real effort task in a large number of experiments, including [11–15]. Performance in this task is typically found to be gender neutral [16]. The three two-digit numbers appeared on the computer screen together with information about whether the answer to the previous exercise was correct and the cumulative number of successfully completed exercises up to that point. The sequence of numbers used in the exercises was randomly generated before the first session of the experiment, so that it was identical for all participants; we used a separate sequence for the baseline phase and the production phase.

Upon entering the laboratory, participants were welcomed and assigned to a random computer. They received the instructions for the baseline phase of the experiment on screen; the instructions included a single comprehension question. After everyone had finished the instructions, the baseline phase started. In the baseline phase, participants worked individually for four minutes and were paid 10 Euro cents for every correct answer they provided. An English translation of all instructions and two screenshots can be found in the Appendix; the original Dutch version of the instructions is available upon request.

### Production phase

After the baseline phase the experiment moved to the production phase, for which participants received additional instructions and comprehension questions. After all participants had finished the instructions and comprehension questions, the production phase started. In this phase, there were three different treatments. In two treatments (BaseProd for **Base**line **Prod**uctivity and ContempProd for **Contemp**oraneous **Prod**uctivity), participants were randomly grouped into teams of four. They were told that as a team they had to solve a number of exercises somewhere between 750 and 1150. Participants were not told the actual number (which was 829) in order to mitigate possible focal number effects. Participants received a fixed fee of 10 Euros for their participation in the production phase, regardless of the number of exercises they had solved individually. There was no fixed time limit; rather, participants were told that they would be paid and could leave after *their team* had finished. If a participant gave an incorrect answer to an exercise, she moved on to the next exercise. Incorrect answers did not contribute to the production of the team, and hence do not count towards a participant’s output.

Note that the incentives are different in the baseline (piece rate) and production phase (time incentives). Nevertheless, the results from workers in the experiment who are not being observed (or observe) suggest that the number of exercises solved in the baseline is a good measure of productivity with time incentives, see the section on positive and negative peer effects below.

**Fig 1:**
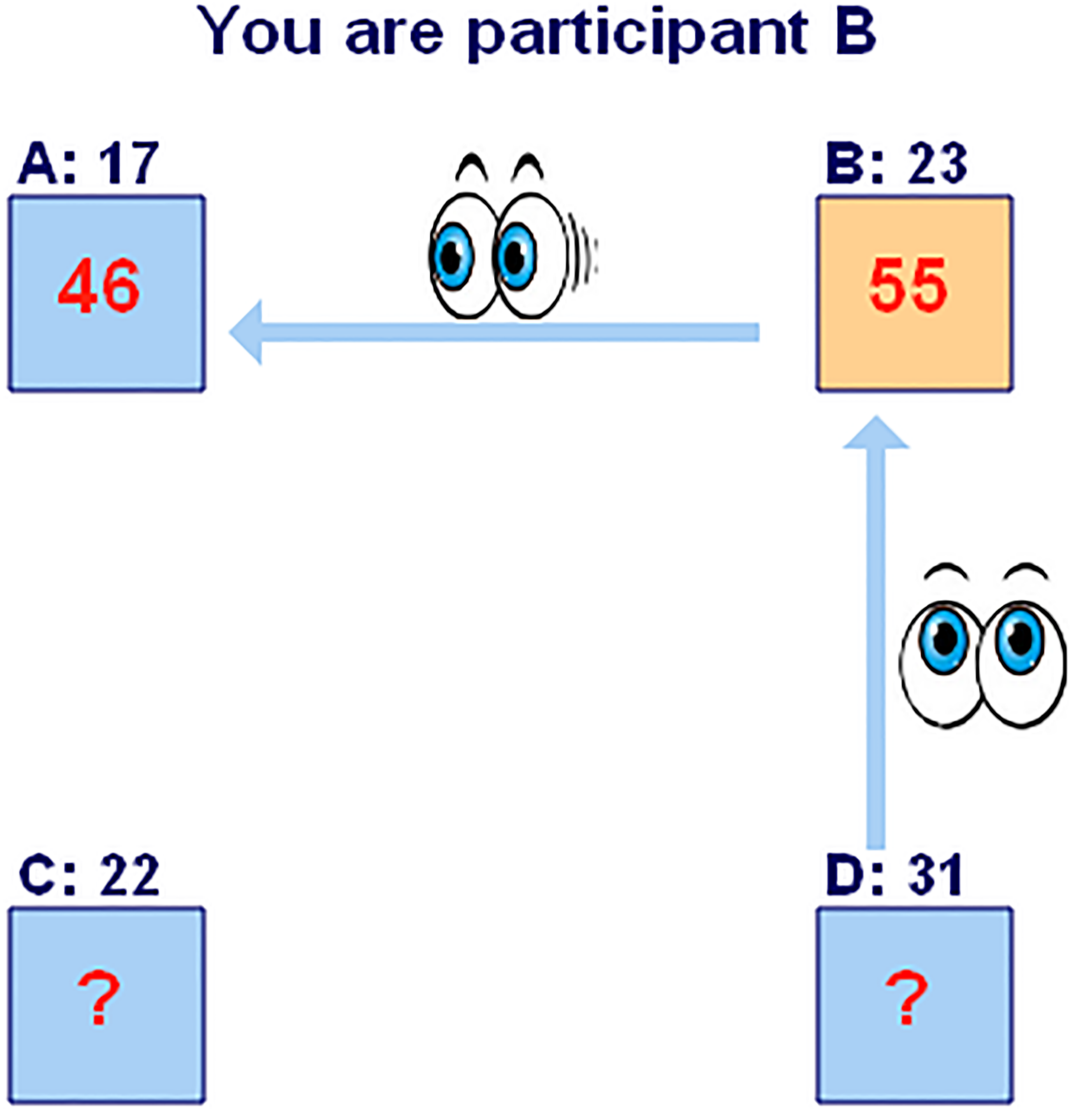
Team overview (Treatment BaseProd). *Notes*. The figure gives the team overview used in treatment BaseProd. The numbers above the squares are the number of exercises solved in the baseline phase (**Base**line **Prod**uctivities). These are visible for all team members in treatment BaseProd and are absent in treatment ContempProd. The numbers inside the squares are the current cumulative number of exercises solved in the production phase (**Contemp**oraneous **Prod**uctivities); these numbers are continuously updated throughout the production phase and available in both treatments. Each participant only knows her own cumulative production and the cumulative production of participants she can see (as indicated by the arrows).

Workers were also told that during the production phase they might receive information about the number of exercises solved by one or more of their teammates in the production phase. In line with [3], we refer to this information as participants’ contemporaneous productivity. The left part of workers’ computer screen contained an overview of their team. Fig 1 gives the team overview used for treatment BaseProd. An arrow going from one participant to another indicates that this participant could see the number of exercises solved by the other in the production phase up to that point. For example, participant B could see that participant A had so far solved 46 exercises.

The team structure we used provides within-session variation in whether a participant could observe the cumulative number of addition problems solved by one of their team members, or could be observed by one of their team members. Specifically, participant A knew the number of exercises he solved could be seen by one team member, whereas participant D knew he could see the number of exercises solved by one team member. Participant B knew he could both see one team member and be seen by another team member and participant C knew he could neither see nor be seen by another participant. The structure of the team remained fixed for the duration of the experiment.

The difference between treatments BaseProd and ContempProd is that participants in treatment BaseProd *also* learned the total number of exercises solved by each of their team members in the *baseline* part of the experiment. They learned the baseline productivity for all participants in their team, even for those for whom they did not know the number of exercises solved in the production phase. For example, all participants in Fig 1 could see that participant C had solved 22 exercises in the baseline. As a consequence, treatment BaseProd allows peer effects to work through baseline productivity as well as contemporaneous productivity. Treatment ContempProd only allows peer effects to work through **Contemp**oraneous **Prod**uctivity. This treatment therefore provides a stronger test of our hypotheses by allowing us to measure if peer effects appear even when workers do not know the exact baseline productivity of their co-workers (as is likely the case in many applications). Note that there is no reflection problem [1] in either treatment, since information flows go only in one direction: for example worker B can be influenced by the contemporaneous productivity of worker A but not vice versa.

Finally, we also ran an individual treatment (treatment NoPeers) in which participants individually were told they had to solve between 188 and 288 exercises (the actual number was 207). Participants in treatment NoPeers never got any feedback about the performance of other participants in the experiment and were allowed to leave the experiment after they had solved the required number of exercises. We included this treatment to check if organizing workers into teams per se changed their productivity.

Note that while our design is somewhat similar to that of [4], there are some important differences: i) their design does not include a player who observes and is also observed; ii) the productivity of observers is unknown to the observed player; iii) participants are paid a piece-rate; iv) observing players are not simultaneously working on the task and v) the way participants are observed is very different. Observers directly observe the actions of the observed player, which allows them to learn how to better approach the task from the observed player. All peer effects [4] find appear in the first few periods, which suggests that their study can tell us more about peer effects on learning than on effort.

### Remaining procedures

For each participant, the production phase ended after she (NoPeers) or the team (BaseProd and ContempProd) had completed the required number of exercises. After finishing their final exercise, participants received an overview of their earnings and were asked to fill out a questionnaire. The questionnaire contained several demographic questions, a self-monitoring questionnaire [17] and questions about the experiment. After finishing the questionnaire, participants could collect their payment and leave the laboratory, even if other participants were still solving exercises or working on the questionnaire.

In total we ran 4 sessions for treatment ContempProd and 4 sessions for treatment BaseProd (N = 84 participants each), as well as 1 session for treatment NoPeers (N = 20). Participants were recruited by sending an email announcement to people who are registered in the participant database of the CREED laboratory for experimental economics at the Faculty of Economics and Business of the University of Amsterdam. Everyone registered in this database has given his/her consent for data from experiments in which they participate to be used for research purposes.

Participants’ average age was 22.5, 38% of participants reported they studied economics and 58% were male. Total earnings consisted of a 7 Euro show-up fee, a 10 Euro fixed fee for the production phase and 10 cents per exercise solved in the baseline phase. Participants earned between 17.60 and 22.50 Euro, with an average of 19.45 Euro. Sessions lasted between 45 and 90 minutes. The average number of exercises solved per minute did not differ significantly between the individual treatment (6.85) and the two team treatments (6.61), so we will focus on the two team treatments in the analysis.

### Testing hypotheses H1 to H3

We can test all three hypotheses formulated in the Introduction using data from treatment BaseProd. H1 (positive peer effects) can be tested by regressing participants’ effort on the average number of exercises solved by their co-workers in the baseline phase (that is, their co-workers’ baseline productivity). Here, we define effort as the number of exercises solved per minute in the production phase, divided by the number of exercises solved per minute in the baseline. This captures the intuition that workers who work faster than in the baseline are expending more effort. Note that we could not directly use the total output (i.e., number of exercises solved) as our measure of effort. This is because participants who are matched with slower co-workers mechanically have to solve more exercises in our experiment, leading to a spurious negative peer effect estimate.

H2 (observability) can be tested by separately regressing participants’ effort in the production phase on the baseline productivity of the co-workers that observe them and the productivity of the co-workers that they observe. Finally, H3 (ability) can be tested by regressing effort in the production phase on average baseline co-worker productivity, separately for participants with low and high baseline productivity.

Stronger tests of hypotheses H1 and H3 can be performed using the data from treatment ContempProd. Although baseline productivity is not observed in this treatment, workers B and D observe the contemporaneous productivity of A and B, respectively. They could infer the baseline productivity of this team member from his contemporaneous productivity. H2 cannot be tested with the data from the ContempProd treatment because observed workers have no information about either baseline or contemporaneous productivity of the workers who observe them.

In practice peer effects may also be negative in the sense that a worker decreases his production speed when matched with a highly productive worker, for example as the result of free riding. If both negative and positive peer effects are present, the average estimated peer effect may therefore be zero. At the end of the results section we therefore present an alternative analysis that allows us to detect peer effects even when negative and positive peer effects are both present in the data.

## Results

We can only expect to find peer effects if participants in the experiment are aware of the information they are supposed to observe and if the task in the experiment allows for sufficient variation in workers’ production speeds. The first subsection therefore presents evidence indicating that these two requirements are satisfied. We then turn to the main results of the paper, where we first estimate the linear-in-means model to examine the presence of peer effects and then investigate whether peer effects differ by observability and workers’ ability. Finally, we use an alternative estimation technique that allows us to provide an estimate of overall peer effects even when the strength and direction of peer effects vary across workers.

### Awareness and variation

Participants can only respond to their co-workers’ productivity if they are aware of how high it is. To assess whether this requirement is fulfilled, we asked participants to recall or guess the number of exercises solved by each of their co-workers in the production phase and (in treatment BaseProd) the baseline phase. These questions were part of the questionnaire, not incentivized and were not announced until after the experiment had ended.

Table 1 presents the correlations between workers’ guesses/recollections and the actual number of solved exercises in the production phase (that is, the contemporaneous productivity). When workers were asked to recall the number of exercises solved by the workers they observed (the bold entries), they did very well in both treatments: the correlations range from 0.56 to 0.94 and are always significantly different from zero at the 1% level.

**Table 1:** Correlations between guessed and actual contemporaneous productivity.

ContempProd	Actual contemporaneous productivity of worker			
	A	B	C	D
Worker A’s guess		0.18	-0.45**	-0.27
Worker B’s guess	0.88***		-0.40*	-0.03
Worker C’s guess	0.00	-0.34		-0.06
Worker D’s guess	-0.09	0.56***	-0.00	
BaseProd	Actual contemporaneous productivity of worker			
	A	B	C	D
Worker A’s estimate		-0.05	-0.14	0.10
Worker B’s estimate	0.94***		0.60***	0.31
Worker C’s estimate	-0.29	-0.24		0.56**
Worker D’s estimate	-0.17	0.93***	0.77***	

*Note*. 6 workers (1 in ContempProd, 5 in BaseProd) did not fill out the respective questions in the questionnaire and were thus omitted from the sample, leaving 162 observations overall.

* *p* < 0.10.

** *p* < 0.05.

*** *p* < 0.01.

The remaining entries in the table show that, logically, workers in treatment ContempProd were unable to guess the number of exercises solved by co-workers they could not observe; correlations range from -0.45 to 0.18. In treatment BaseProd, some workers were able to infer the production phase output of their co-workers from knowing the baseline productivity of these co-workers, and correlations therefore range from -0.29 to 0.77.

Table 2 presents the results for baseline productivity (the number of exercises solved in the baseline phase). The table shows that workers in BaseProd were well aware of the the baseline productivity of their co-workers. All correlations are positive, large and significant at the 1% level. Overall, Tables 1 and 2 suggest that workers were well aware of both the baseline productivity and (when observable) the production phase output of their co-workers.

**Table 2:** Correlations between recalled and actual baseline productivity.

Actual baseline productivity of worker				
BaseProd	A	B	C	D
Worker A’s recollection		.82***	.48**	.71***
Worker B’s recollection	.96***		.94***	.83***
Worker C’s recollection	.85***	.87***		.82***
Worker D’s recollection	.70***	.78***	.89***	

*Note*. 4 workers did not fill out the recall questions and were thus omitted from the sample, leaving 80 observations.

* *p* < 0.10.

** *p* < 0.05.

*** *p* < 0.01.

Second, workers can only respond to information about the productivity of co-workers if they are able to adjust their production speed. We therefore examine whether workers were able to change their production speed across exercises. We do not look directly at the time spent per exercise, since some exercises were more difficult than others. Instead, we normalize the amount of time a worker spent per exercise by dividing it by the average time spent on the exercise among all workers. Table 3 shows that the average standard deviation of the normalized worker production speed is around 40-50% of the average production speed, which suggests that workers had sufficient scope to adjust their production speed in response to peer effects. In addition, we observed several workers who simply stopped working for a few minutes, suggesting that downward adjustments in the production speed were possible as well. Note also that neither the standard deviation nor the weighted standard deviation differs significantly across treatments (p>0.10 for all pairwise treatment comparisons, t-tests).

**Table 3:** Variation in production speed.

Standard deviation of production speed	.451	.495	.425	.372
Weighted standard deviation of production speed	.412	.436	.402	.350
Sample	All	BaseProd	ContempProd	NoPeers
Observations	188	84	84	20

*Notes*. The numbers in the first row are computed in the following way. First, for every worker, we record the amount of time (in milliseconds) spent on each exercise. We then divide this time by the average amount of time spent on the respective exercise by all workers. We then take the standard deviation of this measure for each worker (over all exercises solved), and report the average estimated standard deviation across all workers. For the second row, the procedure is similar, except that the number is a weighted average where each standard deviation is weighted by the inverse of the average production speed of the respective worker.

In addition, to be able to estimate peer effects there also needs to be sufficient between-subject variation in co-worker productivity. Reassuringly, the number of exercises solved in the baseline phase ranged from 6 to 55, with an average of 24 and a standard deviation of 8 exercises.

### Peer effects

Hypothesis H1 states that workers’ effort is increasing in the average baseline productivity of their co-workers. Recall that as our measure of effort we use the average number of exercises solved per minute in the production phase, divided by the number of exercises solved per minute in the baseline to correct for differences in ability. Alternatively, we could have included baseline productivity as a control variable in the regressions. This does not change the conclusions. Average production speed in the experiment was 6.64 exercises per minute; production speed was similar across roles and treatments.

As our measure of co-worker baseline productivity, we take the number of exercises solved by the co-worker in the baseline phase (this is the measure that workers saw on their screen in treatment BaseProd). For treatment BaseProd, we initially take the average baseline productivity of all co-workers as the independent variable; for treatment ContempProd we take the baseline productivity of observable co-workers. For treatment ContempProd, we only included those workers who actually observed a co-worker in the regression (i.e., workers B and D).

Table 4 shows the result of an OLS regression of the log of the focal worker’s effort (i.e., the production speed relative to the baseline) on the log of average co-worker baseline productivity. The log-log specification results in coefficients that can be interpreted as elasticities. The results in the first column are based on the two team treatments together. The point estimate is negative but not significantly different from zero, hence we find no support for hypothesis H1 (positive peer effects). The results in columns (2) and (3) for the separate treatments are very similar. We also ran the regressions without using log transformations, which yielded identical conclusions. We also ran alternative specifications where we replaced average co-worker productivity with maximum, minimum or median productivity; these estimates also resulted in identical conclusions.

**Table 4:** Peer effects estimates.

Dependent Variable: Log worker effort			
	(1)	(2)	(3)
Log average co-worker baseline productivity	-0.059 (0.080)	-0.017 (0.138)	-0.090 (0.100)
Constant	0.206 (0.143)	0.126 (0.242)	0.271 (0.185)
Sample	all	BaseProd	ContempProd B&D
Observations	126	84	42

*Notes*. This table displays the results of three OLS regressions; the numbers in parentheses are robust standard errors.

* *p* < 0.10.

** *p* < 0.05.

*** *p* < 0.01.

### Observability

While we find no evidence that peer effects are relevant at the aggregate level, it is possible that this obscures the fact that peer effects are active more locally. According to hypothesis H2, peer effects should be larger with respect to co-workers who can observe the focal worker. Table 5 displays the results of two regressions that examine if this is the case. The regressions examine if the focal worker’s effort is affected by the baseline productivity of the observing co-worker or the observable co-worker. Since the only treatment in which co-worker baseline productivity is visible to participants is BaseProd, we use only data from this treatment for these regressions.

**Table 5:** Peer effects estimates by observability.

Dependent Variable: Log worker effort		
	(1)	(2)
Log baseline productivity (observing set)	-0.061 (0.124)	
Log baseline productivity (observable set)		-0.094 (0.132)
Constant	.238 (0.214)	0.232 (0.226)
Sample:	BaseProd A&B	BaseProd B&D
Observations	42	42

*Notes*. This table displays the results of two OLS regressions; the numbers in parentheses are robust standard errors.

* *p* < 0.10.

** *p* < 0.05.

*** *p* < 0.01.

Column (1) shows that increasing the baseline productivity of the *observing* co-worker by 10% *decreases* the effort of the focal worker by a not statistically significant 0.61%. Increasing the baseline productivity of *observable*co-workers decreases the effort of the focal worker by 0.94%, here too the coefficient is not statistically different from zero. These results are at odds with hypothesis H2 which stated that a worker’s effort is higher when she is observed by a productive co-worker.

### Ability

Thus far we have found no evidence of peer effects at the aggregate level or separately for observing or observable co-workers. By hypothesis H3, one reason for the lack of effect could be that peer effects only appear among low productivity workers. To investigate if this is the case, we re-estimate the regression of Table 4 separately for low and high productivity workers (using a median split on baseline productivity).

Table 6 shows the results of these regressions. Increasing the average baseline productivity of co-workers by 10% increases the effort of high productivity workers by 2.09%, whereas it reduces the effort of low productivity workers by 0.81%. Thus, if anything high productivity workers appear more likely to be positively affected by peer effects, although neither the coefficients nor the difference in coefficients are significant at conventional levels.

**Table 6:** Peer effects estimates by ability.

Dependent Variable: Log worker effort		
	(1)	(2)
Log average co-worker baseline productivity	-0.081 (0.152)	0.209 (0.176)
Constant	0.345 (0.264)	-0.382 (0.304)
Productivity	low	high
Sample	BaseProd	BaseProd
Observations	44	40

*Notes*. This table displays the results of two OLS regressions; the numbers in parentheses are robust standard errors.

* *p* < 0.10.

** *p* < 0.05.

*** *p* < 0.01.

### Positive and negative peer effects

We have seen no evidence of homogeneously positive (or indeed negative) peer effects in either the full sample or in subgroups based on ability or observability. One explanation for this is that workers were not influenced by peer effects. Another explanation is that workers were influenced by peer effects, but peer effects were heterogeneous along dimensions other than ability or observability in ways that canceled out on average. For example, some workers may have responded to increased co-worker production by free riding, whereas others may have been inspired to increase their effort.

To investigate whether our results are due to positive and negative peer effects canceling out on average, we regress a worker’s average number of exercises solved per minute in the production phase on the average number of exercises solved per minute in the baseline. Intuitively, for workers not influenced by peer effects, the two should be highly correlated: fast workers in the baseline are likely still fast in the production phase. Thus, we should observe a high correlation when peer effects cannot play a role, as in treatment NoPeers and for workers C in the other two treatments.

By contrast, when peer effects play a role, two workers who were equally productive in the baseline might decide to work more or less quickly in the production phase in response to their specific co-workers’ production speeds. The correlation between the own baseline productivity and the production speed in the production phase should then be smaller. Crucially, this will be true independent of the direction of the peer effect for the individual workers. As such, examining the correlation between production speed and baseline productivity allows us to establish the existence of peer effects even when positive and negative peer effects cancel out on average.

Notice that one difference between the baseline and production phase is that workers were paid a piece rate in the baseline phase and received a fixed wage in the production phase. Since this is true in all treatments, this could affect the size of the overall correlation but should not generate treatment differences. Note also that the dependent variable in this analysis differs from the dependent variable in Tables 4 to 6. In previous tables we were interested in the effects of co-worker productivity on a worker’s own effort. Here we are interested in the correlation between baseline and contemporaneous productivity and whether this correlation is different when exposed to peers.

Table 7 examines the relevant correlations using regressions. Column (1) uses data from workers who neither observed nor were observed by another worker during the experiment (treatment NoPeers plus workers C from the other treatments). For these workers, baseline productivity explained 77% of the variation in production phase production speeds. This seems intuitive: in absence of peer effects, there is little reason for workers not to work at a similar speed in the production phase and baseline.

**Table 7:** Positive and negative peer effects.

Dependent Variable: Average production speed per minute of the focal worker in the production phase				
	(1)	(2)	(3)	(4)
Baseline Productivity	0.804*** (0.048)	0.851*** (0.067)	0.543*** (0.092)	0.543*** (0.092)
Baseline Productivity X ContempProd (A,B,D)				0.308*** (0.114)
Baseline Productivity X NoPeers&C				0.261** (0.104)
ContempProd (A,B,D)				-1.589** (0.725)
(NoPeers&C)				-1.399** (0.663)
Constant	1.713*** (0.340)	1.523*** (0.448)	3.112*** (0.569)	3.112*** (0.569)
Sample	NoPeers&C	ContempProd:A,B,D	BaseProd:A,B,D	All
R-Squared	0.77	0.70	0.33	0.66
Observations	62	63	63	188

*Notes*. This table displays the results of four OLS regressions; the numbers in parentheses are robust standard errors.

* *p* < 0.10.

** *p* < 0.05.

*** *p* < 0.01.

Column (2) gives the results for workers with possible peer effects in treatment ContempProd. Interestingly, the results are not very different. Baseline productivity still explains most (70%) of the variation in production phase output. This suggests that peer effects played a limited role in this treatment.

However, the results are quite different for treatment BaseProd (column 3). For workers in this treatment, baseline productivity explains only 33% of the variation in production speeds. This is nearly 50 percentage points less than for workers not influenced by peer effects (column 1). The interaction terms in column (4) show that the effect of baseline productivity on production speed is significantly smaller in treatment BaseProd than in either treatment ContempProd or for workers without peer effects.

Finally, it is worth noting that there is no evidence that the importance of peer effects differed by observability. Estimating regression (3) separately for each worker type yields identical R-squared values for workers A (0.38) and D (0.38). Thus, workers seem to have been equally influenced by peer effects regardless of whether they were observing or being observed. Further, the R-squared value for worker B (0.27) is slightly smaller, suggesting that when workers had both an observing and an observable co-worker, peer effects may have become slightly more important overall.

Overall we find evidence suggestive of a substantial peer influence in treatment BaseProd. This implies that the fact that we found no significant peer effect in the previous analysis is due to positive and negative peer effects canceling out on average. By contrast, there is little evidence of peer effects in treatment ContempProd. Therefore, for peer effects to arise in our experiment it seems to have been important for participants to know the baseline productivity of their co-workers.

## Discussion

This paper reports the results of a lab experiment that was designed to increase our understanding of peer effects in the workplace. More specifically, we formulated and tested three hypotheses: i) a worker’s effort increases in the baseline productivity of his or her peers (positive peer effects), ii) a worker’s effort increases in the baseline productivity of peers that can observe her, but not in the baseline productivity of peers she observes, and iii) positive peer effects are larger for low-productivity workers than for high-productivity workers.

Because the second and third hypotheses were motivated by the findings of [3]’s influential paper, our design attempts to capture some essential features of the work setting of the supermarket chain they analyzed. In particular, workers receive a fixed salary and jointly face a fixed workload such that when a co-workers works harder this translates into more leisure (on-the-job) for others. At the same time, our design differs from the field setting studied by [3] in that we concentrate on one particular channel (observability) through which a worker is influenced by the productivity of his co-workers. We concentrate on this channel because this is the channel that is emphasized by [3] and in the studies referring to it (e.g., [18–21]).

We find no support for any of our three hypotheses. Just like almost half of the other studies on peer effects in productivity surveyed by [10], we find no evidence of positive peer effects. This does, however, not imply that the participants in our experiment are unaffected by having co-workers. Further analysis of our data suggests that some participants respond positively to exposure to high-productive peers, while others respond negatively, resulting in an average effect not significantly different from zero. Our results are in contrast to several other laboratory experiments that do find significantly positive average peer effects [6–8]. According to the regressions reported by [10] this difference may be due to these other lab studies compensating participants based on the group output and due to the workers not being perfect substitutes in production.

We also fail to find positive average peer effects when we zoom in on observing co-workers and on low-productivity workers. These latter results may cast doubt on the general applicability of the channels uncovered by [3], or at least their applicability in settings similar to ours. One reason why our results differ from theirs might be that observability in a real-life setting is different from the observability we implemented in the lab. Observability in the real-life setting of [3] may cause shame, whereas due to anonymity observability in the lab may only trigger guilt [22]. The design of our lab experiment does not capture that distinction.

Finally, our analysis also serves as a reminder that peer effects can be difficult to detect in settings where positive and negative peer effects may cancel out on average. This is likely to be the case in team production tasks, but also in other settings where peer effects based on payoff maximization and social preferences have opposite predictions. Our results also demonstrate the usefulness of measuring individual productivity before participants engage in the team production task. This provides us with a way to establish the existence of peer effects despite positive and negative peer effects appearing to cancel each other out in our sample. However, this analysis is somewhat limited by the fact that productivity was not randomly assigned. In future work, it could be useful to explore exogenous differences in productivity, e.g., by randomly varying productivity across sessions.

## Supporting information

S1 File Online appendix. Contains English translations of the experimental instructions, and screenshots of the experimental program.
(PDF)
