# Supplementary material for: Peers at work: Evidence from the lab
Source: PLoS One. 2018 Feb 6;13(2):e0192038. doi: 10.1371/journal.pone.0192038 (PMC5800692; doi:10.1371/journal.pone.0192038)
Supplement: S1 File — Contains English translations of the experimental instructions, and screenshots of the experimental program. (PDF) [file pone.0192038.s001.pdf]

Supplementary Materials for 'Peers at work: Evidence from the lab'

Roel van Veldhuizen   Hessel Oosterbeek   Joep Sonnemans

A Instructions

Welcome to this experiment.[1] During the experiment you are not allowed to communicate with other participants. If you have a question, please raise your hand. One of the experimenters will then come to your cubicle to answer your question.

Today's experiment consists of two parts; part two will take considerably more time than part one. Part two of the experiment will be explained after you have finished part one of the experiment. Your income will be determined on the basis of your results in the experiment. You will also receive a show-up fee of 7 Euros.

Please read through the following instructions carefully. As part of the instructions you will be asked a practice question to test your understanding of the instructions. When you have correctly answered this question, the experiment will move on. Using the navigation bar at the top of your screen it will be possible to return to previous pages during the instructions and practice question.

Instructions Part One

In part one of the experiment the procedure will be as follows. The computer screen will display three two-digit numbers (as in the example below). Your task is to calculate the sum of these three numbers. For every correct answer you will receive 10 Euro cents. An incorrect answer does not earn you any money; after an incorrect answer you will automatically go on to the next exercise. This part of the experiment will take up 4 minutes in total; during these 4 minutes you can do as many exercises as you want. The clock in the lower right corner of the screen tells you how much time you have left. The number of exercises you have answered both correctly and incorrectly is displayed above the current exercise; the (+1) indicates if the previous exercise was answered correctly or incorrectly.

[1] These are an English translation of the instructions for treatment BaseProd. The Dutch originals and the instructions for the other treatments are available on request.

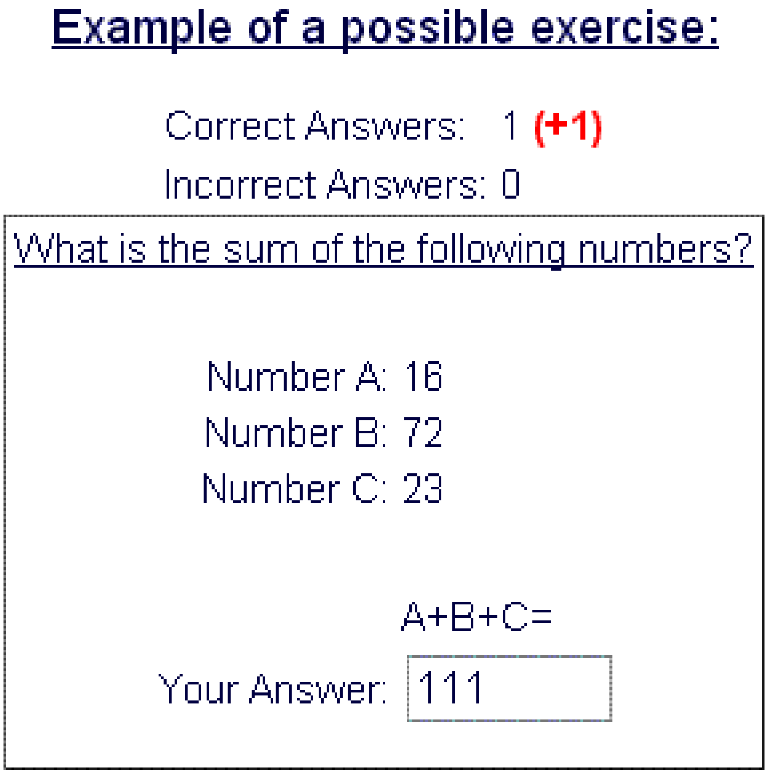

Practice Question

Hank has finished 11 exercises, providing the correct answer to 8 and an incorrect answer to 3. How many Euro cents has Hank earned?

Instructions End

You are now ready for part 1 of the experiment. By pressing the link below you will reach a waiting screen. The first part of the experiment starts as soon as all the others have also finished the instructions. On the waiting screen you can read back the text of the instructions.

Instructions Part Two

Like in part one, your task in part two will be to add three two-digit numbers. However, during this part of the experiment you will form a team with three other persons. The experiment will last until you and the other three people in your team have provided a correct answer to a fixed number of exercises. This fixed number of exercises will be somewhere between 750 and 1150 exercises. For this part of the experiment both you and all other team members will get a fixed payment of 10 Euros.

As soon as you and your team have solved the required number of exercises, the experiment will be over for your team after a short questionnaire. One of the experimenters will come to your cubicle to pay out your earnings for

the experiment. After payment you can leave the laboratory, even if the other teams are not done yet.

Information

During the experiment the left side of your screen provides an overview of your team, comparable to the figure displayed below. Each of the squares A, B, C and D represents one of the team members; your square will be colored in orange (in the example below you are participant B). Within the figure, the blue numbers above the squares indicate how many exercises each participant has solved in part 1 of the experiment. Thus, in the example below, participant A has solved 18 exercises, participant B 22, participant C 35 and participant D 21.

The figure also contains arrows between some participants. In the example below there is an arrow from participant D to participant B, an arrow from participant B to participant A and an arrow from participant A to participant B. These arrows represent information flows. An arrow from one participant to another indicates that this participant is able to see the number of solved exercises of the other participant in part two up to that point. Only the number of correct answers will be counted. The number of solved exercises by other participants will be displayed in red letters within the square that corresponds to said participant.

In the example below you are participant B and can see how many exercises you have solved (117) and how many exercises participant A has solved (125). In the example below you have **no** information about the number of exercises solved by participants C and D, who therefore have a "?" in their corresponding square. This means that you will at no stage get to know the number of exercises solved by participants C and D (not even after the experiment).

Finally, note that both participant A and participant D can see how many exercises you have solved up to that point. Participant C, however, does not know how many exercises you have solved and will at no stage get to know this number (not even after the experiment).

The figure below only represents an example of a who-sees-who; the who-sees-who that will be used in the experiment (which can have fewer, more or different arrows) will be announced after the instructions. However, the who-sees-who will remain the same during the experiment; id est, both the arrows, your participant letter and the participant letter of the other participants will remain the same for the whole of the experiment. The composition of your team will not change during the experiment either.

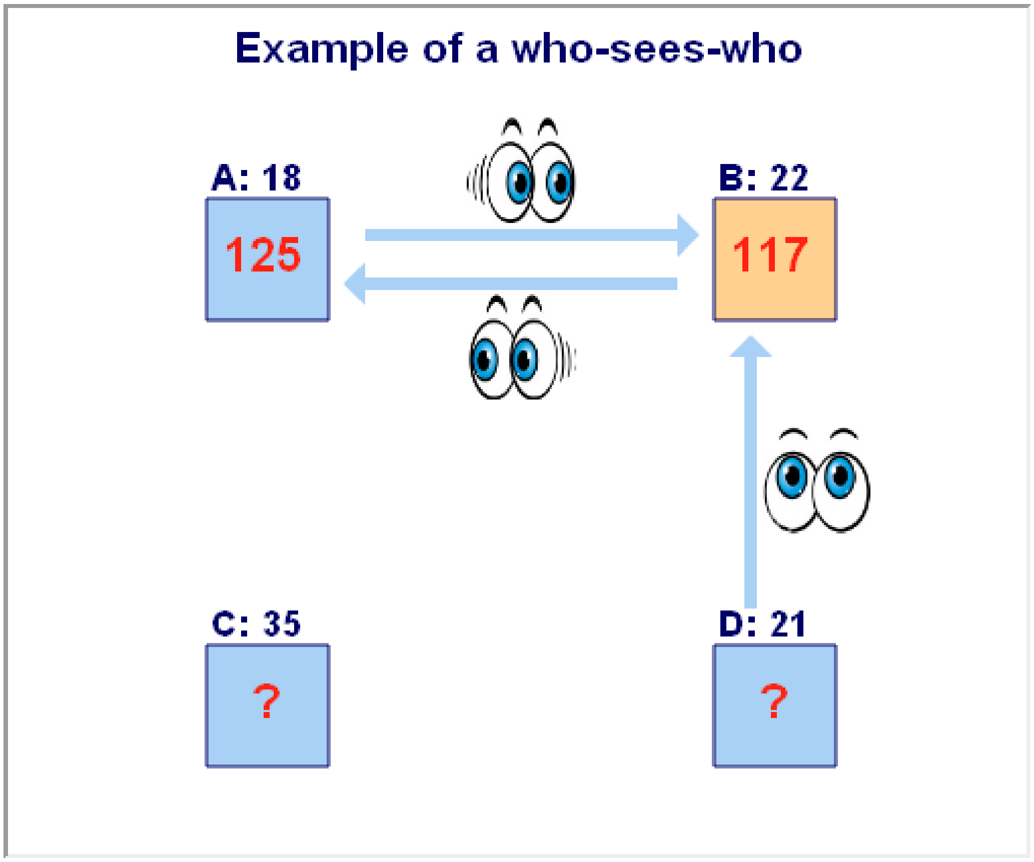
Example of a who-sees-who
A: 18
125
B: 22
117
C: 35
?
D: 21
?

Example Screen

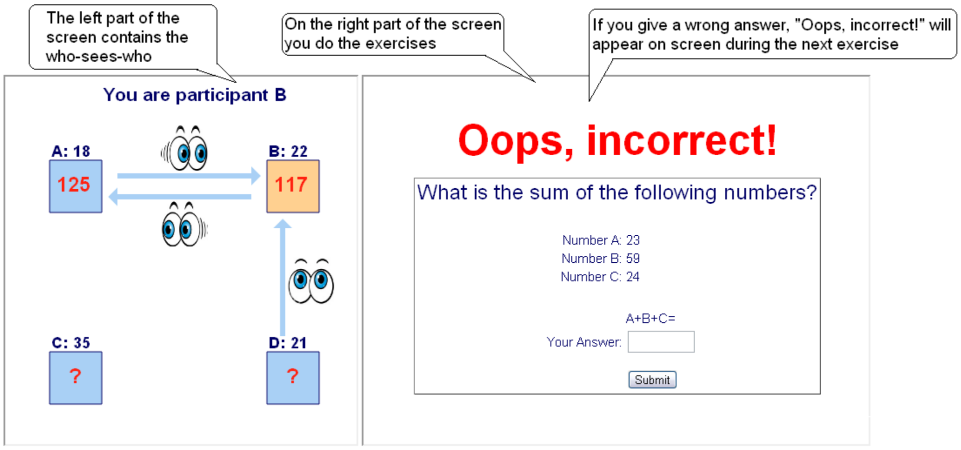

Check-Up Question 1

The figure below gives an example of a who-sees-who. Indicate for every team member for which team member he or she can see the number of solved exercises. Also indicate for every team member who can see the number of exercises solved by them.

	Participant A	Participant B	Participant C	Participant D
Participant **A** knows the number of exercises solved by:	☐	☐	☐	☐
Participant **B** knows the number of exercises solved by:	☐	☐	☐	☐
Participant **C** knows the number of exercises solved by:	☐	☐	☐	☐
Participant **D** knows the number of exercises solved by:	☐	☐	☐	☐
This participant knows the number of exercises solved by participant **A**:	☐	☐	☐	☐
This participant knows the number of exercises solved by participant **B**:	☐	☐	☐	☐
This participant knows the number of exercises solved by participant **C**:	☐	☐	☐	☐
This participant knows the number of exercises solved by participant **D**:	☐	☐	☐	☐

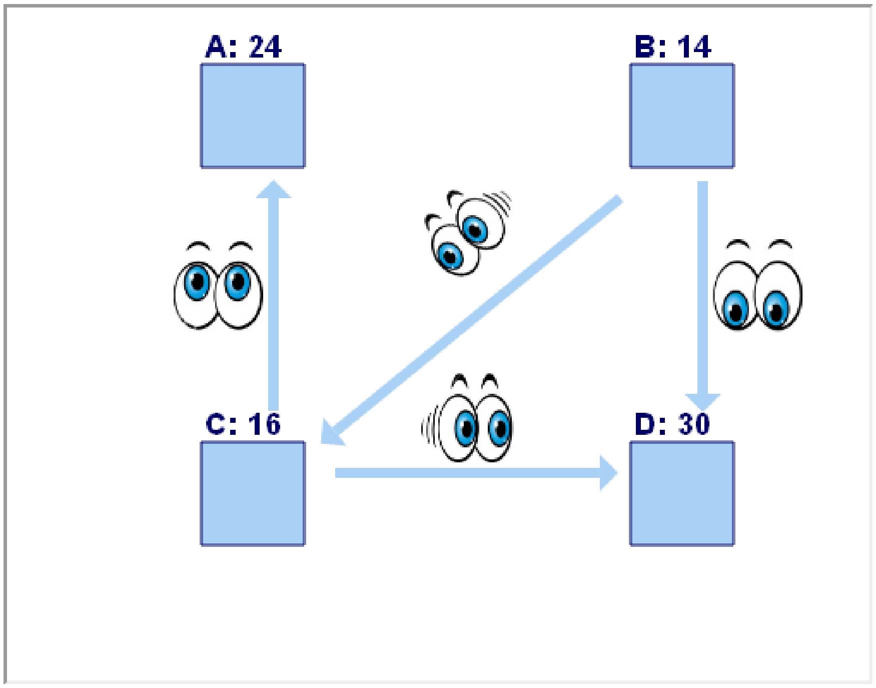

Check-Up Question 2

The figure below gives an example of a who-sees-who (the same one as in the previous question). You are participant C, therefore you have all the information that participant C has access to. Indicate for all participants how many

exercises they have solved in part one of the experiment. When possible, indicate for every participant how many exercises this participant has solved so far in this part of the experiment (part two). If the number of solved exercises is not known, do not fill in anything.

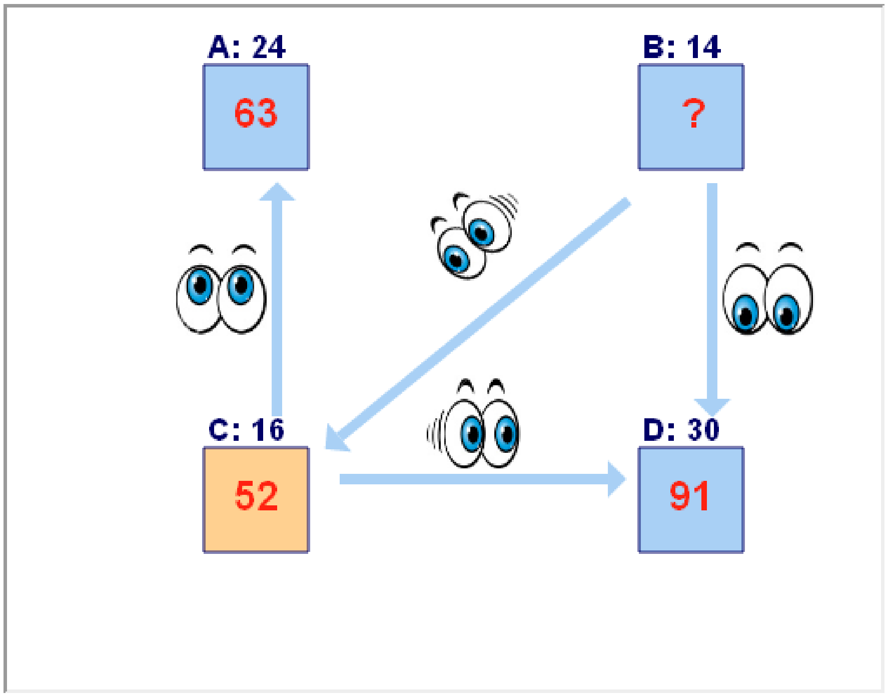

Check-Up Question 3

Finish the sentence: this part of the experiment ends when *you/your team mates/you and your team mates/everybody in the experiment* have given the correct answer to *750/1150/a fixed number between 750 and 1150* exercises.

Check-Up Question 4

Which participants will know after the experiment how many exercises you have answered correctly?

- All participants in the experiment
- All participants who during the experiment could see the number of exercises you solved.
- All participants of which during the experiment you could see the number of exercises solved.
- Nobody

Instructions End

You are now ready to start part two of the experiment. By pressing the link below you will arrive at a waiting screen. Part two of the experiment will start as soon as all participants have finished the instructions. On the waiting screen you can read back the instructions of this part of the experiment as well.

B Screenshots

Figs 1 and 2 are screenshots of the baseline and production phase of the experiment respectively.

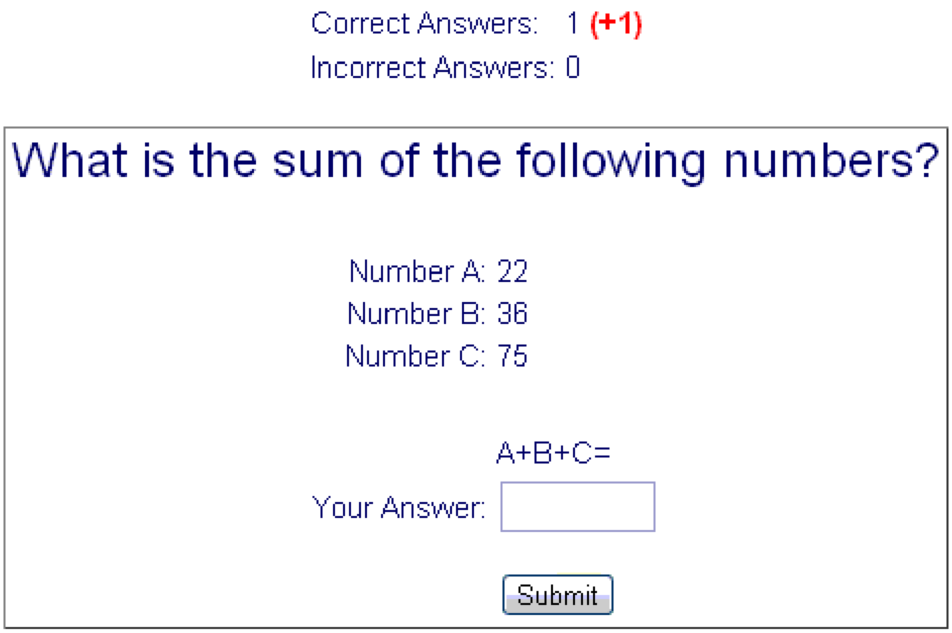

Figure 1: Screenshot of the Baseline Phase

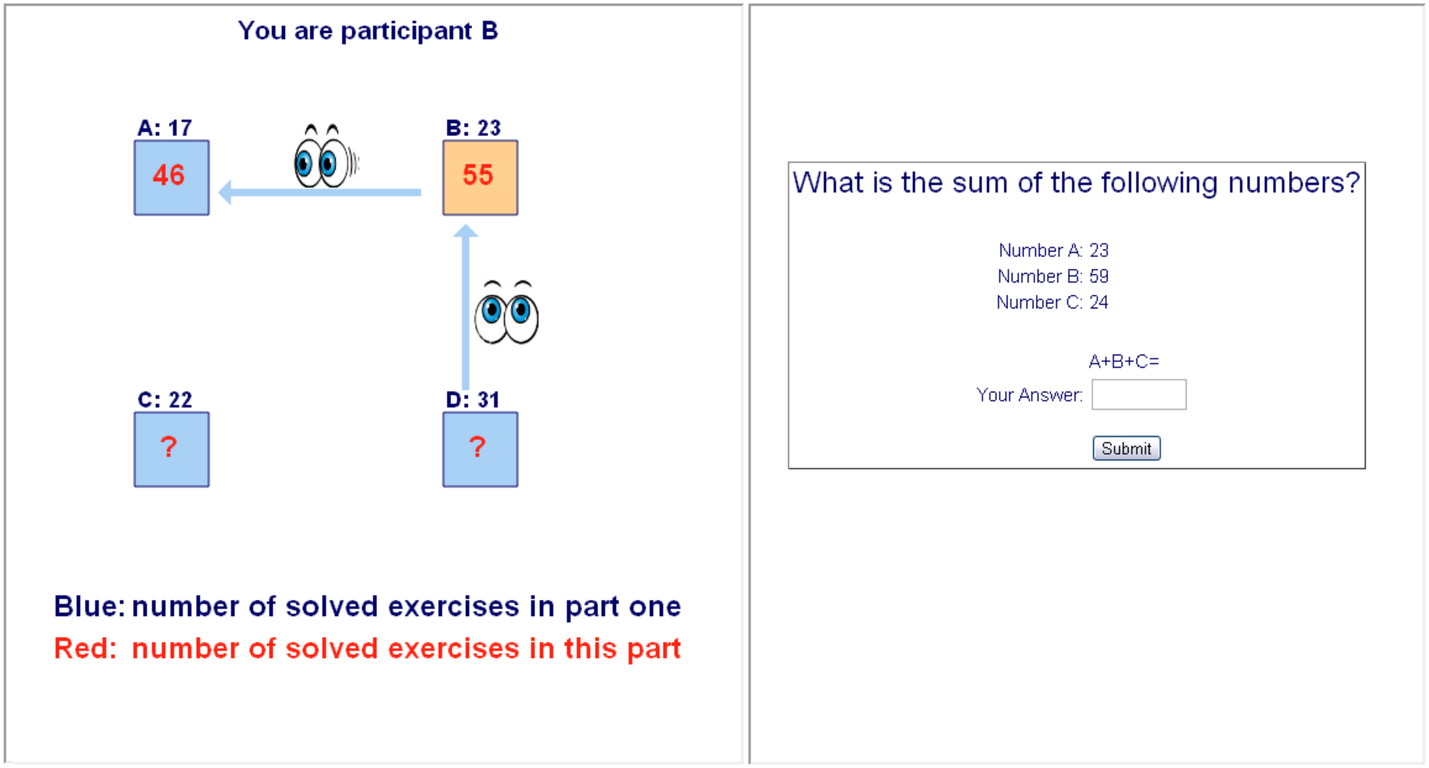

Figure 2: Screenshot of the Production Phase
